# In vivo digestibility of six selected fodder species by goats in northern Ghana

**DOI:** 10.1007/s11250-019-01989-w

**Published:** 2019-07-13

**Authors:** F. K. Avornyo, S. T. Partey, R. B. Zougmore, S. Asare, A. A. Agbolosu, N. M. Akufo, N. A. Sowah, S. P. Konlan

**Affiliations:** 1 Council for Scientific and Industrial Research, Animal Research Institute, Nyankpala Station, Tamale, Ghana; 2 International Crops Research Institute for the Semi-Arid Tropics, BP 320, Bamako, Mali; 3 University for Development Studies, Nyankpala Campus, Tamale, Ghana; 4 Department of Animal Science, University of Ghana, Legon, Accra, Ghana

**Keywords:** Browse species, Digestibility, Supplementation, West African Dwarf goats

## Abstract

An in vivo digestibility trial was conducted to evaluate the digestibility of six forages. These were selected indigenous browse species and groundnut haulms in Lawra and Jirapa Districts of the Upper West Region of Ghana. Groundnut haulms served as a control due to its known good digestibility in small ruminants. Thirty West African Dwarf (WAD) young castrated billy goats with a mean age of 8 months were used. The average live body weight was 8.9 ± 0.1 kg. The animals were randomly assigned to the six treatments and replicated five times in a completely randomized design. They were confined in metabolism cages. The adaptation period was 3 weeks after which data was taken for 10 days. The treatments were the leaves and tiny twigs of T1 *Annona senegalensis*, T2 *Ficus gnaphalocarpa*, T3 *Pericopsis laxiflora*, T4 *Pterocarpus erinaceus*, T5 *Afzelia africana*, and T6 (control) *Arachis hypogaea*. Feed intake, faecal matter and urine output were measured. The results indicated that dry matter (DM) contents of the feedstuffs were less than 50% but crude protein (CP) contents were higher than the minimum required for sustaining ruminants. Neutral and acid detergent fibre contents were high, and *F. gnaphalocarpa*, in particular, contained a higher level of phosphorus (P). The amounts of feed intakes were typical of most fodder species with the exception of *P. laxiflora* and *P. erinaceus*, which were lower (*P* < 0.05). The apparent digestibility values were also typical of most fodder species but the digestibility of *A. africana* fibre appeared to be lower. Nitrogen retention was positive for all test species with the exception of *P. laxiflora*, which was negative. Mortality was recorded in animals on *P. laxiflora*, and the rate was high. *Pericopsis laxiflora* leaves, even though morphologically similar to the leaves of *P. erinaceus*, were not a suitable fodder. *Ficus gnaphalocarpa* appeared to be the overall best fodder species in terms of nutrient uptake.

## Introduction

In Ghana, livestock production employs over 60% of rural households in the three northern regions (Martey et al. 2012). Investment in this industry is critical for mitigating poverty and enhancing food security. The rainfall pattern in this part of Ghana is unimodal, and the area constitutes the major livestock production hub of the country. The climate is characterized by a dry season of about 6-month duration every year where there is a drastic decline in both quality and quantity of forage (Otchere et al. 2002). Free-ranging ruminants usually put on weight in the rainy season due to abundance of quality and nutritive feed, whereas their condition, particularly those of cattle and sheep, may drastically deteriorate as pasture abundance and quality generally decline towards the end of the dry season. The nutritive value of the predominant pasture species in the dry season is very poor with an average crude protein (CP) content of less than 7%, and grazing livestock may be deficient in about 50% of their required CP intake (Khan and Habib 2012).

Ruminants are sometimes offered supplementary feed which may consist of cereal and leguminous crop residues as well as agroindustrial byproducts and some selected browse species (Karikari et al. 1995). Low-quality forage-based diets invariably impede their productivity due to a lower dry matter (DM) intake, high-fibre content and lower digestibility and nutritive value of ingested feed. It is established that supplementation of CP, minerals and energy-rich feeds increases microbial fermentation of low-quality fibrous feeds in the rumen that helps maximize total DM intake and improves animal productivity (Khan et al. 2009; Patra 2010). In the predominantly small-scale, subsistence farming systems in the northern part of Ghana, most of the farmers cannot afford a continuous supplementation of concentrate feeds to their animals. Recent research is therefore directed towards the exploration of an affordable and abundant, alternate CP and energy-rich feeds from the prevalent evergreen tree species available (Osuga et al. 2005).

Findings show that tree leaves can be more efficiently utilized as a low-cost CP and mineral supplement to low-quality fibrous diets in the tropics, particularly during the prolonged feed scarcity periods (Patra 2010). In this regard, tree leaves are receiving increasing attention due to their potential to supply high CP and minerals and palatable fodder during periods of reduced accessibility to good-quality fodder (Abebe et al. 2012; Khan and Habib 2012). It is in view of the above that this study was conducted to assess the digestibility of some indigenous tree and shrub species that farmers in Jirapa and Lawra Districts in the Upper West Region of Ghana have prioritized for supplementary feeding of their goats.

## Materials and methods

### Experimental location

The experiment was carried out at the Nyankpala Station of the Animal Research Institute of the Council for Scientific and Industrial Research. Nyankpala is located on longitude 1° 4′ W and latitude 9° 26′ N and at a height of about 183 m above sea level and in the dry savanna ecological zone in the Tolon District of the Northern Region. It has a unimodal rainfall pattern that begins in May and ends in October. The mean annual rainfall is about 1043 mm. Temperatures normally fluctuate between 15 °C (minimum) and 42 °C (maximum) with a mean annual temperature of about 28.3 °C. The mean annual daytime relative humidity is about 54%. The Nyankpala area experiences dry cold Harmattan winds from November to February and a period of warm dry conditions from March to April. The dry season therefore stretches from November to April (GSS 2014).

### Experimental design, animals and treatments

The experimental setup was a completely randomized design. Thirty faecal bags were sewn for the collection of faecal matter. Five sets of six metabolism cages were constructed and fitted with slatted floors for the passage of faeces that fail to enter the bag, and urine into separate receptacles. The side nets of the cages were electro galv. welded wire mesh made in Accra, Ghana. The faecal collection mesh was made from fibre netting.

Thirty West African Dwarf young billy goats of the same age and body weight were procured. Their mean age was approximately 8 months and mean live body weight was 8.9 ± 0.1 kg at the start of the experiment. Prior to the beginning of the experiment, the animals were ear-tagged; given antibiotics, ivermectin and vitamins (100 ml Kepro oxytet 20% LA inj. (oxytetracycline), Kepro B.V., Holland; 200 ml oxytetravet aerosol by Arab pesticides and vet drugs mfg., Co., Jordan; Ivermectin, Kepromec 50 ml by Kepro B.V., Holland, info@kepro.nl; 100 ml Introvit, made by Interchemie werken, Holland; Latex medical examination gloves made in Malaysia; 10 ml Uni-ject syringes and needles, Quadact Limited, UK); and castrated to facilitate identification and data management. These treatments were performed to clear the experimental animals of any bacterial and parasitical infections, and standardize their health condition prior to the commencement of the experiment. Previous studies (Avornyo et al. 2015) have shown evidence of the effectiveness of the medicines used. The goats were individually housed in metabolism cages measuring 60 cm by 70 cm and 70 cm high, and equipped with feeding and water troughs. Six fodder species including a control were used for the experiment. They were as follows: T1, *Annona senegalensis*; T2, *Ficus gnaphalocarpa*; T3, *Pericopsis laxiflora*; T4, *Pterocarpus erinaceus*; T5, *Afzelia africana*; and T6, *Arachis hypogaea* (control) (Table 1). They were harvested up to a distance of 15-km radius from the site of the experiment. The feeds offered contained leaves and twigs less than 3 mm in diameter. The animals were randomly assigned to the treatments with each experimental treatment offered to five individual animals, each representing a replicate. Water and mineral lick (Selena blocks—Na 38%; Ca 1%; Mg 0.5%; ZnO 290 mg/kg; MnSO_4_ 180 mg/kg; Ca 40 mg/kg; cobalt acetate 28 mg/kg and NaSe 6 mg/kg; NaCl 95.5%; CaCO_3_ 7.7%; and calcined magnesia 1%, www.nutriblock.com, Spain) were provided ad libitum. Each animal was given the treatment two times a day at about 9 a.m. and 2 p.m. Slight adjustments were made to the amount of feed offered during the period of adaptation to the feed such that feed left over was between 10 and 20%. The amount of feed given on dry matter basis was therefore maintained at about 3% of the experimental animal’s live body weight. The animals were solely on the experimental feeds. The containers for collecting voided urine was injected with 20 ml of 5% H_2_SO_4_ daily and connected under the metabolism cages for urine collection.

**Table 1:** Characteristics and general uses of the fodder species used in the experiment

Scientific name	Common name	Family	Characteristics	Climate range	Uses	References
*Afzelia africana*	African mahogany	Caesalpinioideae	Deciduous multipurpose tree	Senegal to Sudan and south to Congo	Bark Stem/branches Seeds for medicine Fuel wood, Game tool (oware); fodder, soil fertility improvement, fuelwood	Avornyo et al. (2018); Husseini et al. (unpublished)
*Ficus gnaphalocarpa*	Mulberry fig	Moraceae	Semi-deciduous multipurpose tree	Drier areas from Senegal to Ethiopia, south to S. Africa, east to the Arabian Peninsula and Madagascar	Fruits Leaves Bark for food Fodder Medicine, soil fertility improvement, fuelwood, shade	Fern et al. (2014); Avornyo et al. (2018); Husseini et al. (unpublished)
*Annona senegalensis*	African custard apple	Annonaceae	Deciduous multipurpose multi-stemmed shrub	Senegal to Kenya, south to Zimbabwe	Fodder, medicine, food	Fern et al. (2014); Avornyo et al. (2018)
*Arachis hypogaea*	Groundnut	Fabaceae	Annual plant with erect or prostrate stems up to 70 cm long	Tropics to the warm temperate zone	Food, medicine, fodder	Fern et al. (2014)
*Pericopsis laxiflora*		Fabaceae	Deciduous shrub or tree with a dishevelled crown of crooked, drooping branches	Senegal to Sudan	Roots, bark and leaves for medicine	Fern et al. (2014)
*Pterocarpus erinaceus*	African kino	Fabaceae	Deciduous multipurpose tree	Sierra Leone to Central African Republic	Fodder, medicine, for making xylophone; fuelwood, soil fertility improvement, shade	Fern et al. (2014); Avornyo et al. (2018)

### Data collection and analysis

A preliminary feeding period of 21 days was followed by 10 days of data collection. Daily records of feed and water intakes as well as leftovers were taken. All the leftovers of each feed offered on each day were pooled per treatment per day, thoroughly mixed and resampled for analysis daily. The same procedure was used for taking faeces and urine samples. Ten percent of faecal material was sampled per treatment per day. One hundred millilitres (100 ml) of urine was also sampled daily per treatment and stored at 4 °C. Representative samples of the feeds offered, leftover and faecal matter were stored frozen. Dry matter was determined by oven drying at 60 °C for 48 h (AOAC 1990). Feed, refusals and faeces were analysed for dry matter (DM), crude protein (CP), organic matter (OM), neutral detergent fibre (NDF), acid detergent fibre (ADF), acid detergent lignin (ADL), calcium (Ca) and phosphorus (P) (AOAC 1990; Van Soest et al. 1991). Urine was analysed for nitrogen (N) (AOAC 1990).

### Chemical analysis

Chemical analysis was done in the nutrition laboratory of the Animal Science Department of the University of Ghana, to determine the DM, CP, ash, Ca and P by AOAC (1990) method. Neutral detergent fibre, ADF and ADL were determined according to Van Soest et al. (1991).

### Data analysis

Data collected were analysed using the General Linear Model procedure of SAS 9.4 edition for Windows (SAS 2013). Where results were significant, least significant difference was used for mean comparison at 5% probability level.

## Results

### Chemical composition of experimental fodder species

The DM contents of the browse plants were in the range of 280.0 to 450.2 gkg^−1^ with *P. laxiflora* having the highest and *A. hypogaea* having the least DM content (Table 2). The CP contents of the browse plants were highest for *A. africana* (136.7 gkg^−1^ DM) and least for *A. senegalensis* (81.2 gkg^−1^ DM). *Pericopsis laxiflora* also had the highest NDF content while *A. hypogaea* recorded the lowest. *Pericopsis laxiflora* also had the highest ADF content while *P. erinaceus* recorded the lowest ADF value (*P* < 0.05). Calcium and ADL contents were not significantly different among the browse plants. While *F. gnaphalocarpa* recorded the highest P content, *A. senegalensis* had the lowest value (*P* < 0.05).

**Table 2:** Chemical composition of the leaves of experimental fodder species (gkg^−1^ DM except for DM which is gkg^−1^ fresh leaves)

Parameter	Foliage type						SEM	*P* value
	*A. africana*	*F. gnaphalocarpa*	*A. senegalensis*	*A. hypogaea*	*P. laxiflora*	*P. erinaceus*		
DM	406.9^b^	363.5^e^	380.5^c^	280.0^f^	450.2^a^	376.7^d^	2.35	0.0001
CP	136.7 ^a^	115.9^b^	81.2^d^	90.4^cd^	102.4^bc^	105.3^bc^	7.04	0.0001
Ash	106.6^c^	241.4^a^	68.6^e^	185.2^b^	115.5^c^	86.5^d^	3.58	0.0002
NDF	645.0^ab^	669.3^a^	604.1^ab^	578.7^b^	675.8^a^	617.1^ab^	19.1	0.0195
ADF	400.5^ab^	506.0^ab^	545.5^ab^	510.0^ab^	575.5^a^	348.9^b^	49.4	0.0322
ADL	129.2	146.0	173.1	86.8	102.8	85.7	25.4	0.1427
Ca	12.1	12.4	15.3	16.3	13.0	12.3	2.71	0.1780
P	1.00^bc^	3.19^a^	0.22^d^	1.01^bc^	0.84^c^	1.35^b^	0.21	0.0001

Row values with different superscript letters are significantly different at *P* < 0.05

*DM*, dry matter; *CP*, crude protein; *NDF*, neutral detergent fibre; *ADF*, acid detergent fibre; *ADL*, acid detergent lignin; *Ca*, calcium; *P*, phosphorus

### Nutrient intake and digestibility

**Fig. 1:**
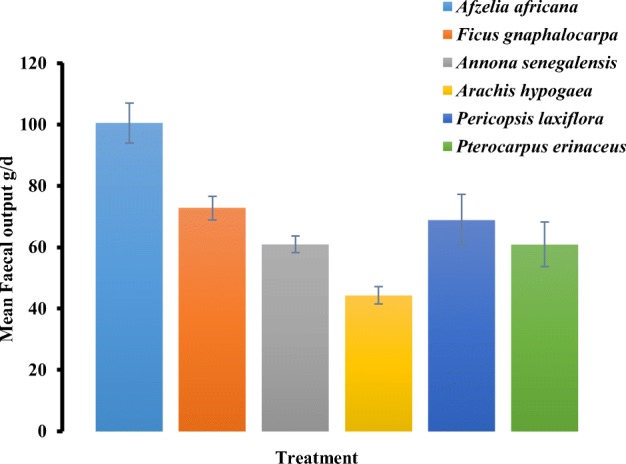
Mean faecal output of young billy goats fed six different fodder species

The highest DMI (264.9 gd^−1^) was recorded in *F. gnaphalocarpa* whereas the least DMI was recorded in *P. erinaceus* (191.8 gd^−1^) (*P* < 0.05). Dry matter intake was about 3% of live body weight for four feeds and about 2% for *P. erinaceus* and *P. laxiflora* (Table 3). There was a highly significant difference (*P* < 0.001) in the total faecal output among the treatments. *Afzelia africana* recorded the highest faecal output (100.5 gd^−1^) whereas *A. hypogaea* recorded the least (44.3 gd^−1^) (Fig. 1).

**Table 3:** Nutrient intake, apparent digestibility and N balance in West African Dwarf goats fed six fodder species (*n* = 10; edf = 45)

Parameter	Fodder type						SEM	*P* value
	*A. africana*	*F. gnaphalocarpa*	*A. senegalensis*	*A. hypogaea*	*P. laxiflora*	*P. erinaceus*		
Nutrient intake								
DMI, gd^−1^	242^b^	264.9^a^	238.8^b^	227.1^b^	208.1^c^	191.8^c^	19.7	0.0001
DMI, gkg^−1^ LW	28.4^b^	34.1^a^	28.2^b^	29.9^b^	23.4^c^	23.1^c^	2.06	0.0001
N intake, gd^−1^	5.43^a^	4.95^b^	3.23^c^	3.39^c^	3.18^c^	3.46^c^	0.49	0.0001
Urine N, gd^−1^	2.06^b^	1.14^d^	1.06^d^	1.46^cd^	3.07^a^	1.77^bc^	0.05	0.0001
Faecal N, gd^−1^	1.59^a^	1.17^b^	1.23^b^	0.82^c^	1.04^bc^	0.85^c^	0.28	0.0001
Apparent digestibility (%)								
DM	53.3^ab^	69.1^a^	69.5^a^	73.9^a^	62.8^a^	41.8^b^	16.3	0.0005
OM	52.0^c^	63.7^abc^	71.6^ab^	75.5^a^	60.7^bc^	54.4^c^	11.1	0.0001
CP	70.8^a^	76.3^a^	61.3^b^	75.2^a^	60.5^b^	71.9^a^	7.63	0.0001
NDF	46.7	70.4	62.3	67.5	60.1	60.6	19.8	0.1484
ADF	28.1	64.2	67.1	68.1	64.3	53.5	38.6	0.1854
Nitrogen balance								
N retention, gd^−1^	1.78^b^	2.64^a^	0.94^c^	1.11^c^	− 0.94^d^	0.84^c^	0.58	0.0001
N ret. %N intake	33.2^ab^	53.3^a^	28.7^ab^	32.8^ab^	− 41.1^c^	21.7^b^	21.2	0.0001
N ret. %N digested	46.5^a^	70.0^a^	45.6^a^	43.1^a^	− 73.5^b^	28.2^a^	37.9	0.0001

Values are the means of 10 replicates. Values in row with different superscripts are significantly different at 5% probability level

*DMI*, dry matter intake; *LW*, live weight; *N*, nitrogen; *DM*, dry matter; *OM*, organic matter; *CP*, crude protein; *NDF*, neutral detergent fibre; *ADF*, acid detergent fibre; *N ret*., nitrogen retention

The highest N intakes were observed in *A. africana* followed by *F. gnaphalocarpa* before the remaining four fodder species (*P* < 0.05). Urine N output was high in *P. laxiflora* but lower (*P* < 0.05) in the remaining species particularly in *A. senegalensis* and *F. gnaphalocarpa*. Faecal N was higher in *A. africana* but lowest in *P. erinaceus* and *A. hypogaea* (*P* < 0.05).

Dry matter digestibility was around 60 to 75% for four of the feeds but around 42 to 53% for *A. africana* and *P. erinaceus* (*P* < 0.05). Organic matter digestibility was 75.5% in *A. hypogaea* which was significantly higher (*P* < 0.05) than 52%, 54% and 61% observed in *A. africana*, *P. erinaceus* and *P. laxiflora*, respectively. *Annona senegalensis* and *F. gnaphalocarpa* had values closer to that of *A. hypogaea*. On the contrary, crude protein digestibility (CPD) was higher in *F. gnaphalocarpa*, *A. hypogaea*, *P. erinaceus* and *A. africana* than in *A. senegalensis* and *P. laxiflora* (*P* < 0.05). Significant differences were not observed in NDF and ADF digestibility.

Nitrogen retention was highest in *F. gnaphalocarpa* followed by *A. africana* and least in *P. laxiflora* (*P* < 0.05) which was the only species associated with negative N retention. Of the *F. gnaphalocarpa* N consumed, only a little over half was assimilated. It was about one-third in the other species excluding *P. laxiflora*. Of the *F. gnaphalocarpa* N digested, about 70% of it was assimilated but this value was not significantly higher than the values obtained for the other species excluding *P. laxiflora*.

## Discussion

### Chemical composition and digestibility

The CP contents of the experimental feeds were higher than the minimum range of 60–80 gkg^−1^ DM required for sustenance of microbial growth (Norton 1994; Ansah 2015). However, they were lower than those reported by Abebe et al. (2012) and Salem et al. (2015) for some browse species. The species considered in this study generally had lower CP values compared with those studied by Osuga et al. (2005) in Kenya. Ash contents of *F. gnaphalocarpa* and *A. hypogaea* were higher than values obtained by Abebe et al. (2012) and Gemeda and Hassen (2015) but similar to values obtained by Osuga et al. (2005). *Ficus gnaphalocarpa* exudes a sticky substance when cut, and *A. hypogaea* haulm is uprooted from the soil and is therefore likely to be more contaminated with soil, hence the high ash contents. Fibre values observed in this study were higher than those of some browse species but not grass species reported by Abebe et al. (2012). There were somewhat higher values of NDF, ADF, Ca and P, and lower of CP content observed for *F. gnaphalocarpa* in this study than that of Yahaya et al. (2001). However, Salem et al. (2006) observed high-fibre contents in some of the browse species they studied. Agroecological, species and seasonal and age differences might have accounted for some of these variations (Devendra 2012). This notwithstanding, nutritional values of *A. senegalensis* reported by Meale et al. (2012) were fairly similar to those reported in this study.

Total DMI was within the expected 3% of animal live body weight in the case of *A. senegalensis*, *F. gnaphalocarpa*, *A. africana* and *A. hypogaea* but low for *P. laxiflora* and *P. erinaceus* suggesting that these two species may not be adequate as sole feeds for goats. Gidado et al. (2013) reported saponin level of *P. erinaceus* to be 4.42%, which would impart a bitter taste (Reddy 2001). This amount was higher than ruminant-tolerable level of 1.5 to 2% reported by Onwuka (1983). On the contrary, *P. erinaceus* was a popular browse plant in northern Ghana, and it was the most common fodder species sold in the Upper West Region (Avornyo et al. unpublished). Drying of fodder prior to its feeding however might improve palatability (Reddy 2001). Moreover, browsing activity under free range may be different from the same activity under confinement. Also, there is much similarity in the appearance of the leaves of *P. laxiflora* and *P. erinaceus* and so it is easy to mistake one for the other. The low consumption of *P. laxiflora* might also be related to its content of anti-nutritive factors. It might be that *P. laxiflora* contained high levels of organic substances such as tannin (Degen et al. 2010) and/or other substances that impart an offensive odour or bitter taste (Harbourne 1993). *Pericopsis laxiflora* was not regarded as fodder plant in northern Ghana, but because of its similarity to *P. erinaceus*, it was identified more frequently as a preferred fodder species by farmers than *Combretum molle*, which was next in a list of preferred fodder species identified by farmers (Avornyo et al. 2018). Although the fodder species were not analysed for tannin content, we suspect that the presence of tannins in feedstuffs might have reduced the rate of consumption (Salawu et al. 1997; Yisa et al. 2010). A study that involved the inclusion of Moringa leaf meal in a diet for small ruminants was associated with DMI of between 4 and 5% (Jiwuba et al. 2016). Even though Bruinenberg et al. (2003) did not observe a clear relationship between digestibility and DMI, higher DMIs appeared to be associated with higher digestibility in this study.

By inference, the intake of ash in *P. hypogaea* and *F. gnaphalocarpa* would be high as compared with the other fodder species. According to Parr (1988), the upper limit of recommended range of ash intake is 10%. Similarly, the intake of P in *F. gnaphalocarpa* would be relatively higher compared with the other treatments. Nitrogen intake appeared to be satisfactory in almost all the fodder species especially *F. gnaphalocarpa* and *A. africana*.

Apparent digestibility values were within the normal range for recommended fodder species; however, apparent digestibility of NDF and ADF of *A. africana* was quite low. As observed by Tolera et al. (1997), Toure et al. (1998) and Gemeda and Hassen (2015), high levels of tannins and polyphenols may be responsible for lowering feed degradability. Observed digestibility of browse undertaken by Toure et al. (1998) appeared lower than in this study, and this might indicate the extent to which goats used in this study might have adapted to the consumption of browse as compared with sheep used in that study. The OM digestibility values of *A. senegalensis* and *A. hypogaea* appeared to be higher than those of *Medicago sativa* hay, which is considered to be of good nutritive value (Ramirez-Orduna et al. 2003), but similar to that of an intensively managed grassland silage in a digestibility study conducted by Bruinenberg et al. (2003). Digestibility values of extensively managed grassland silages were however lower than the digestibilities observed in this study (Bruinenberg et al. 2003) with the exception of *P. erinaceus* and *A. africana* whose values were comparable. The digestibility values appeared similar to those obtained by Sabia et al. (2015) for berseem clover but higher than those obtained for barley by the same authors. Digestibility tends to be higher in species with higher protein content but lower content of fibre and phytochemicals (Abebe et al. 2012). Observations by Sabia et al. (2015) also support the notion that digestibility is negatively affected by ADF content of feed. Apparent digestibility values of *F. gnaphalocarpa*, *A. hypogaea* and *A. senegalensis* appeared to be superior despite relatively high contents of ADL in *F. gnaphalocarpa* and *A. senegalensis*. Physical characteristics of fodder leaves may influence their digestibility (Jung and Allen 1995). Yahaya et al. (2001) found that DM and CP digestibilities of *Ficus polita* and *Acacia sieberina* were higher than that of *F. gnaphalocarpa* but the nominal values were lower than those observed for *F. gnaphalocarpa* in this study. According to Kumara Mahipala et al. (2009), offering some browse species as sole feed may increase crude protein digestibility but offering it at about 50% supplement to a basal hay diet may optimize the digestibility of the other nutrients. Some browse species also have the capacity to replace concentrate supplements in an attempt to achieve the same production target (Assefa et al. 2008); however, goats on *P. erinaceus* and *P. laxiflora* appeared to show visual signs of intoxication towards the latter stages of the experiment.

Nitrogen intakes were higher in goats that consumed *A. africana* and *F. gnaphalocarpa*; however, higher urine N losses were associated with *A. africana* and *P. laxiflora*. Nitrogen retention was therefore superior in animals that consumed *F. gnaphalocarpa* but also high in the consumption of *A. africana*. Retention of N when *A. senegalensis* or *P. erinaceus* was consumed was comparable with *A. hypogaea* when consumed. Generally, about half of the N in these forage species is not utilized by goats but may rather be present in their urine and faeces. Bruinenberg et al. (2003) recovered about two-thirds of N consumed in the faeces and urine of dairy cows. Ruminant faeces and urine may therefore constitute valuable nutrient sources for crops.

The study has indicated that with the exception of *P. laxiflora*, the other browse species had digestibility indices that were within the normal range for recommended fodder species such as groundnut haulm. Considering daily intake values, *P. erinaceus* may also not be suitable as a sole feed to meet maintenance level of requirement but may be suitable as a feed supplement. *Ficus gnaphalocarpa* appeared to be the overall best fodder species in terms of nutrient uptake.
